# Supplementary material for: End Sequence Analysis Toolkit (ESAT) expands the extractable information from single-cell RNA-seq data
Source: Genome Res. 2016 Oct;26(10):1397–410. doi: 10.1101/gr.207902.116 (PMC5052061; doi:10.1101/gr.207902.116)
Supplement: Supplemental Material [file supp_gr.207902.116_Supp_Materials.tar.gz › ESAT-esat_v0.1/doc/html/classbroad_1_1pda_1_1seq_1_1clip_1_1_sample_data.html]

ESAT: broad.pda.seq.clip.SampleData Class Reference


ESAT


- Main Page
- Related Pages
- Packages
- Classes
- Files

- Class List
- Class Index
- Class Hierarchy
- Class Members

All Classes Namespaces Files Functions Variables Enumerator Pages

- broad
- pda
- seq
- clip
- SampleData

Public Member Functions |
Static Public Attributes |
Protected Attributes |
List of all members

broad.pda.seq.clip.SampleData Class Reference

Inheritance diagram for broad.pda.seq.clip.SampleData:

[legend]

Collaboration diagram for broad.pda.seq.clip.SampleData:

[legend]

|  |  |
| --- | --- |
| Public Member Functions | |
|  | SampleData (String bamFile, boolean firstReadTranscriptionStrand, Map< String, Collection< Gene >> genes, double expressionCutoff, boolean expByScanPval, boolean fullyContained) throws IOException |
|  | |
| int | hashCode () |
|  | |
| void | setExpressionScanPvalueCutoff (double expressionScanPvalCutoff) |
|  | |
| double | getExpressionScanPvalueCutoff () |
|  | |
| double | getGeneCount (Gene gene) |
|  | |
| double | getFragmentLengthGeneCount (Gene gene) |
|  | |
| double | getGeneScanPval (Gene gene) |
|  | |
| double | getGeneAverageCoverage (Gene gene) |
|  | |
| boolean | isExpressed (Gene gene) |
|  | |
| boolean | firstReadTranscriptionStrand () |
|  | |
| double | getEnrichmentOverGene (Gene gene, Annotation window) |
|  | |
| Logger | getLogger () |
|  | |
| String | getSampleName () |
|  | |
| String | getOriginalBamFile () |
|  | |
| void | setSampleName (String name) |
|  | |
| TranscriptomeSpaceAlignmentModel | getData () |
|  | |
| TranscriptomeSpaceAlignmentModel | getFragmentLengthFilterData () |
|  | |

|  |  |
| --- | --- |
| Static Public Attributes | |
| static Logger | logger = Logger.getLogger(SampleData.class.getName()) |
|  | |

|  |  |
| --- | --- |
| Protected Attributes | |
| String | sampleName |
|  | |
| TranscriptomeSpaceAlignmentModel | data |
|  | |
| TranscriptomeSpaceAlignmentModel | maxFragmentLengthData |
|  | |
| Map< Gene, ScanStatisticScore > | maxFragmentLengthGeneScores |
|  | |
| Map< Gene, ScanStatisticScore > | geneScores |
|  | |
| Map< Gene, Double > | geneAvgCoverage |
|  | |
| Map< String, Collection< Gene > > | genesByChr |
|  | |
| Map< String, Gene > | genesByName |
|  | |
| double | expressionCutoffValue |
|  | |
| boolean | expressionByScanPval |
|  | |
| boolean | fullyContainedReads |
|  | |

## Detailed Description

Author
:   shari

## Constructor & Destructor Documentation

|  |  |  |  |
| --- | --- | --- | --- |
| broad.pda.seq.clip.SampleData.SampleData | ( | String | *bamFile*, |
|  |  | boolean | *firstReadTranscriptionStrand*, |
|  |  | Map< String, Collection< Gene >> | *genes*, |
|  |  | double | *expressionCutoff*, |
|  |  | boolean | *expByScanPval*, |
|  |  | boolean | *fullyContained* |
|  | ) |  | throws IOException |

Parameters
:   |  |  |
    | --- | --- |
    | bamFile | Bam file |
    | firstReadTranscriptionStrand | Whether read 1 is in direction of transcription |
    | genes | Genes by chromosome |
    | window | Window size |
    | step | Step size |
    | expressionCutoff | P value cutoff for scan test of gene expression |
    | expByScanPval | Expression is assessed by scan P value. If false, uses average read depth |
    | fullyContained | Count fully contained reads only |

Exceptions
:   |  |  |
    | --- | --- |
    | IOException |  |

Here is the call graph for this function:

## Member Function Documentation

|  |  |  |  |  |
| --- | --- | --- | --- | --- |
| boolean broad.pda.seq.clip.SampleData.firstReadTranscriptionStrand | ( |  | ) |  |

Whether read 1 is in direction of transcription

Returns
:   True iff first read transcription strand is true

Here is the caller graph for this function:

|  |  |  |  |  |
| --- | --- | --- | --- | --- |
| TranscriptomeSpaceAlignmentModel broad.pda.seq.clip.SampleData.getData | ( |  | ) |  |

Write the window scores to a file for future use

Parameters
:   |  |  |
    | --- | --- |
    | m | Multi sample binding site caller that this belongs to |

Exceptions
:   |  |  |
    | --- | --- |
    | IOException | Get the alignment data |

Returns
:   Alignment model

|  |  |  |  |
| --- | --- | --- | --- |
| double broad.pda.seq.clip.SampleData.getEnrichmentOverGene | ( | Gene | *gene*, |
|  |  | Annotation | *window* |
|  | ) |  |  |

Get enrichment of a window over a gene

Parameters
:   |  |  |
    | --- | --- |
    | gene | The gene |
    | window | Window contained in the gene |

Returns
:   Enrichment of window over gene background

Here is the call graph for this function:

|  |  |  |  |  |
| --- | --- | --- | --- | --- |
| double broad.pda.seq.clip.SampleData.getExpressionScanPvalueCutoff | ( |  | ) |  |

Get genome wide scan P value cutoff for expression of transcript

Returns
:   P value cutoff for transcript expression against genomic background

|  |  |  |  |  |
| --- | --- | --- | --- | --- |
| TranscriptomeSpaceAlignmentModel broad.pda.seq.clip.SampleData.getFragmentLengthFilterData | ( |  | ) |  |

Get the alignment data filtered by max fragment length

Returns
:   Alignment model

|  |  |  |  |  |  |
| --- | --- | --- | --- | --- | --- |
| double broad.pda.seq.clip.SampleData.getFragmentLengthGeneCount | ( | Gene | *gene* | ) |  |

Get number of fragments mapping to gene after fragment length filter Get score from cache or calculate and cache score

Parameters
:   |  |  |
    | --- | --- |
    | gene | The gene |

Returns
:   The number of fragments mapping to the gene

|  |  |  |  |  |  |
| --- | --- | --- | --- | --- | --- |
| double broad.pda.seq.clip.SampleData.getGeneAverageCoverage | ( | Gene | *gene* | ) |  |

Get average coverage of gene Get score from cache or calculate and cache score

Parameters
:   |  |  |
    | --- | --- |
    | gene | The gene |

Returns
:   The average coverage of the gene

Here is the caller graph for this function:

|  |  |  |  |  |  |
| --- | --- | --- | --- | --- | --- |
| double broad.pda.seq.clip.SampleData.getGeneCount | ( | Gene | *gene* | ) |  |

Get number of fragments mapping to gene Get score from cache or calculate and cache score

Parameters
:   |  |  |
    | --- | --- |
    | gene | The gene |

Returns
:   The number of fragments mapping to the gene

|  |  |  |  |  |  |
| --- | --- | --- | --- | --- | --- |
| double broad.pda.seq.clip.SampleData.getGeneScanPval | ( | Gene | *gene* | ) |  |

Get coordinate space wide scan P value of number of fragments mapping to the gene Get score from cache or calculate and cache score

Parameters
:   |  |  |
    | --- | --- |
    | gene | The gene |

Returns
:   The scan P value of the number of fragments mapping to the gene with respect to teh coordinate space

|  |  |  |  |  |
| --- | --- | --- | --- | --- |
| Logger broad.pda.seq.clip.SampleData.getLogger | ( |  | ) |  |

Get the logger

Returns
:   The logger

|  |  |  |  |  |
| --- | --- | --- | --- | --- |
| String broad.pda.seq.clip.SampleData.getOriginalBamFile | ( |  | ) |  |

Get name of original bam file

Returns
:   Bam file name

|  |  |  |  |  |
| --- | --- | --- | --- | --- |
| String broad.pda.seq.clip.SampleData.getSampleName | ( |  | ) |  |

Get the sample name

Returns
:   Sample name

|  |  |  |  |  |
| --- | --- | --- | --- | --- |
| int broad.pda.seq.clip.SampleData.hashCode | ( |  | ) |  |

|  |  |  |  |  |  |
| --- | --- | --- | --- | --- | --- |
| boolean broad.pda.seq.clip.SampleData.isExpressed | ( | Gene | *gene* | ) |  |

Whether the gene is significantly expressed in the sample

Parameters
:   |  |  |
    | --- | --- |
    | gene | The gene |

Returns
:   Whether the gene is expressed at the given significance level

Here is the call graph for this function:

|  |  |  |  |  |  |
| --- | --- | --- | --- | --- | --- |
| void broad.pda.seq.clip.SampleData.setExpressionScanPvalueCutoff | ( | double | *expressionScanPvalCutoff* | ) |  |

Set genome wide scan P value cutoff for expression of transcript

Parameters
:   |  |  |
    | --- | --- |
    | expressionScanPvalCutoff | P value cutoff for transcript expression against genomic background |

|  |  |  |  |  |  |
| --- | --- | --- | --- | --- | --- |
| void broad.pda.seq.clip.SampleData.setSampleName | ( | String | *name* | ) |  |

Set the sample name

Parameters
:   |  |  |
    | --- | --- |
    | name | Sample name |

## Member Data Documentation

TranscriptomeSpaceAlignmentModel broad.pda.seq.clip.SampleData.data

protected

boolean broad.pda.seq.clip.SampleData.expressionByScanPval

protected

double broad.pda.seq.clip.SampleData.expressionCutoffValue

protected

boolean broad.pda.seq.clip.SampleData.fullyContainedReads

protected

Map<Gene, Double> broad.pda.seq.clip.SampleData.geneAvgCoverage

protected

Map<String, Collection<Gene> > broad.pda.seq.clip.SampleData.genesByChr

protected

Map<String, Gene> broad.pda.seq.clip.SampleData.genesByName

protected

Map<Gene, ScanStatisticScore> broad.pda.seq.clip.SampleData.geneScores

protected

Logger broad.pda.seq.clip.SampleData.logger = Logger.getLogger(SampleData.class.getName())

static

TranscriptomeSpaceAlignmentModel broad.pda.seq.clip.SampleData.maxFragmentLengthData

protected

Map<Gene, ScanStatisticScore> broad.pda.seq.clip.SampleData.maxFragmentLengthGeneScores

protected

String broad.pda.seq.clip.SampleData.sampleName

protected

---

The documentation for this class was generated from the following file:

- java/broad/pda/seq/clip/SampleData.java


---

Generated on Fri Aug 15 2014 15:11:02 for ESAT by  

 1.8.7
